# Clinical characterization of children and adolescents with *NF1* microdeletions

**DOI:** 10.1007/s00381-020-04717-0

**Published:** 2020-06-12

**Authors:** Hildegard Kehrer-Sawatzki, Lan Kluwe, Johannes Salamon, Lennart Well, Said Farschtschi, Thorsten Rosenbaum, Victor-Felix Mautner

**Affiliations:** 1 Institute of Human Genetics, University of Ulm and University of Ulm Medical Center, Albert-Einstein-Allee 11, 89081 Ulm, Germany; 2 Department of Maxillofacial Surgery, University Medical Center Hamburg-Eppendorf, Hamburg, Germany; 3 Department of Neurology, University Medical Center Hamburg-Eppendorf, Hamburg, Germany; 4 Department of Diagnostic and Interventional Radiology and Nuclear Medicine, University Medical Center Hamburg-Eppendorf, Hamburg, Germany; 5 Department of Pediatrics, Sana Kliniken Duisburg, Duisburg, Germany

**Keywords:** Neurofibromatosis type 1, *NF1* microdeletion, Autism spectrum disorder, Cognitive impairment, Neurofibroma, Attention deficit hyperactivity disorder (ADHD)

## Abstract

**Purpose:**

An estimated 5–11% of patients with neurofibromatosis type 1 (NF1) harbour *NF1* microdeletions encompassing the *NF1* gene and its flanking regions. The purpose of this study was to evaluate the clinical phenotype in children and adolescents with *NF1* microdeletions.

**Methods:**

We retrospectively analysed 30 children and adolescents with *NF1* microdeletions pertaining to externally visible neurofibromas. The internal tumour load was determined by volumetry of whole-body magnetic resonance imaging (MRI) in 20 children and adolescents with *NF1* microdeletions. Furthermore, the prevalence of global developmental delay, autism spectrum disorder and attention deficit hyperactivity disorder (ADHD) were evaluated.

**Results:**

Children and adolescents with *NF1* microdeletions had significantly more often cutaneous, subcutaneous and externally visible plexiform neurofibromas than age-matched patients with intragenic *NF1* mutations. Internal neurofibromas were detected in all 20 children and adolescents with *NF1* microdeletions analysed by whole-body MRI. By contrast, only 17 (61%) of 28 age-matched NF1 patients without microdeletions had internal tumours. The total internal tumour load was significantly higher in *NF1* microdeletion patients than in NF1 patients without microdeletions. Global developmental delay was observed in 28 (93%) of 30 children with *NF1* microdeletions investigated. The mean full-scale intelligence quotient in our patient group was 77.7 which is significantly lower than that of patients with intragenic *NF1* mutations. ADHD was diagnosed in 15 (88%) of 17 children and adolescents with *NF1* microdeletion. Furthermore, 17 (71%) of the 24 patients investigated had T-scores ≥ 60 up to 75, indicative of mild to moderate autistic symptoms, which are consequently significantly more frequent in patients with *NF1* microdeletions than in the general NF1 population. Also, the mean total T-score was significantly higher in patients with *NF1* microdeletions than in the general NF1 population.

**Conclusion:**

Our findings indicate that already at a very young age, *NF1* microdeletions patients frequently exhibit a severe disease manifestation which requires specialized long-term clinical care.

**Electronic supplementary material:**

The online version of this article (10.1007/s00381-020-04717-0) contains supplementary material, which is available to authorized users.

## Introduction

Neurofibromatosis type 1 (NF1) is a hereditary tumour predisposition syndrome with an incidence of 1 in 2000–3000 [1–4]. NF1 belongs to the group of phacomatoses which present with characteristic clinical symptoms of the skin, central and peripheral nervous system as well as tumours. The hallmark features of NF1 are café-au-lait spots, Lisch nodules, neurofibromas and an increased risk for malignant peripheral nerve sheath tumours as well as optic pathway gliomas. NF1 is caused in the majority of patients by mutations located within the *NF1* gene on chromosome 17q11.2. However, 4.7–11% of all NF1 patients harbour large deletions encompassing the *NF1* gene and its flanking regions at 17q11.2 [5–8]. These large *NF1* deletions have also been termed ‘*NF1* microdeletions’. Most frequent are type-1 *NF1* microdeletions which encompass 1.4 Mb and include 14 protein-coding genes as well as four microRNA genes [9–11]. Type-1 deletions account for 70–80% of all large *NF1* deletions and usually occur as germline deletions that are present in all cells of the affected patients [12, 13]. Less frequent than type-1 *NF1* deletions are type-2, type-3 and atypical *NF1* deletions that are distinguishable by their size and breakpoint location, by the number of genes located within the deletion region and the frequency of mosaicism with normal cells without the *NF1* microdeletion [reviewed by 14].

Since the first identification of patients with *NF1* microdeletions, it became clear that these patients often present with a severe clinical phenotype and exhibit features which are not common in patients with intragenic *NF1* mutations including facial dysmorphic features, severe learning disabilities, high numbers of neurofibromas, overgrowth/tall-for-age stature, large hands and feet with excessive soft tissue, hyperflexibility of joints and congenital cardiac anomalies [8, 15–21]. The average intelligence in the group of patients with *NF1* microdeletions was found to be lower than in patients with intragenic *NF1* mutations [18, 22, 23].

Importantly, individuals with *NF1* microdeletions have a lifetime MPNST risk in the range of 16–26% [18, 24], which is higher than the estimated lifetime MPNST risk in the general NF1 population of 8–13% according to Evans et al. [25, 26] or 15.8% according to Uusitalo et al. [27]. Further, MPNSTs occur significantly earlier in patients with *NF1* microdeletions as compared with NF1 patients with intragenic mutations [24].

Several studies suggested that patients with *NF1* microdeletions not only exhibit a high number of cutaneous neurofibromas but also an early (prepubertal) onset of cutaneous neurofibroma growth [9, 17, 28–30]. However, a comprehensive analysis of children has not yet been performed in order to ascertain whether an early (prepubertal) onset in growth of neurofibromas is significantly more prevalent in children with *NF1* microdeletions as compared to children with intragenic *NF1* mutations. Furthermore, the clinical phenotype in very young patients with *NF1* microdeletions pertaining to cognitive abilities, developmental delay and the presence of autistic symptomatology has not been systematically investigated as yet.

In this study, we have addressed these issues and investigated the frequency of cutaneous, subcutaneous and plexiform neurofibromas as well as the internal tumour load in children and adolescents with *NF1* microdeletions. In order to further characterize the clinical phenotype in this patient group, we also investigated cognition, the frequency of facial dysmorphic features, developmental delay, autistic symptoms, attention deficit, heart defects and skeletal anomalies. Our analyses indicate that the clinical phenotype associated with *NF1* microdeletions is frequently very severe already at a young age of the patients who need long-term, specialized clinical care as well as intensive psychological and educational support.

## Patients and methods

### NF1 study population

In this study, we retrospectively characterized the clinical phenotype of 30 children and adolescents with *NF1* microdeletions. The type and the extent of the *NF1* deletions were analysed in our previous studies or in the course of the current study by either MLPA using the SALSA P122 C1 MLPA assay (MRC Holland, Amsterdam, Netherlands) or microarray analysis of blood leucocytes (Supp. Table S1) [31–37].

Eighteen of the 30 patients with *NF* microdeletions analysed were males, and 12 patients were females. One patient was a familial case, and the others were sporadic harbouring de novo *NF1* microdeletions. The median age of the 30 patients was 4 years, and the mean age was 5.9 years (range: 1–15 years) at the time of investigation for the presence of externally visible neurofibromas. Eleven of these 30 patients were investigated for the presence of externally visible neurofibromas also in later years of their life (Supp. Table S2).

Six of the 30 patients with *NF1* microdeletions analysed here have been clinically analysed by us already in a previous study which included a total of 29 patients with *NF1* microdeletions [18]. In addition to the six children, this latter study included also 23 *NF1* microdeletion patients who were mostly adults (mean age: 26.3 years).

### Control group of patients with intragenic *NF1* mutations

We also investigated 30 age-matched children and adolescents with intragenic *NF1* mutations identified and clinically characterized at the Department of Neurology, University Medical Center Hamburg-Eppendorf. The median age of these patients was 5.0 years, and the mean age was 5.7 years (range: 1–15 years). This cohort of patients was analysed with regard to the presence or absence of cutaneous, subcutaneous and externally visible plexiform neurofibromas and used as control for the comparison with the number of patients with *NF1* microdeletions exhibiting these tumours.

### Externally visible neurofibromas

In total, 30 children and adolescents with *NF1* microdeletions were evaluated for the presence of cutaneous, subcutaneous and externally visible plexiform neurofibromas. Cutaneous and subcutaneous neurofibromas were differentiated by the fact that cutaneous tumours move with the skin, whereas the skin can be moved over the top of subcutaneous tumours.

### Internal tumour load

The load of internal neurofibromas of 20 children and adolescents with *NF1* microdeletions was analysed by whole-body MRI performed at 1.5 T (Siemens Magnetom, Siemens Healthineers, Erlangen, Germany) between December 2004 and February 2017. The total volume of internal tumours in millilitres was determined by volumetric analysis of T2-weighted sequences in axial orientation (T2w HASTE TIRM: TR 1200 ms; TE 85 ms, FA 148°; Matrix 384 × 384; FOV 450 × 450mm; slice thickness 8 mm; intersection gap 10 mm) using MedX software (v3.42) as described previously [38]. The internal tumour load observed in the 20 *NF1* microdeletion patients investigated in this study was compared with the internal tumour load of 28 age-matched NF1 patients without large deletions analysed by us in a previous study [39].

### Brain tumours

In total, 29 *NF1* microdeletion patients were analysed by head MRI in order to determine the presence of optic pathway gliomas (OP gliomas) or tumours located anatomically distinct from the optic pathway (non-optic pathway gliomas, non-OP gliomas).

### Developmental delay and cognition

Global developmental delay in motor and language skills was assessed in 30 children with *NF1* microdeletions by means the modified Munich Functional Developmental Diagnostics (Münchener Funktionelle Entwicklungsdiagnostik, MFED) for children at the age of 1–3 years [40, 41].

Cognitive ability in 24 patients with *NF1* microdeletions was assessed at the age of 6–18 years by means of the German language versions of Wechsler Intelligence Scales (Hamburg-Wechsler Intelligence Scales HAWIK-R, HAWIK-III and HAWIK-IV) [42, 43]. Based on these tests, mean full-scale intelligence quotient (FSIQ) values was determined.

### ASD and ADHD

Autism spectrum disorder (ASD) symptoms were assessed in 24 patients with *NF1* microdeletions at the age of 4–18 years by means of the Social Responsiveness Scale (SRS) [44]. This questionnaire includes 65 items rated on a 4-point Likert scale (from 1 = never true to 4 = almost always true). It provides information concerning social awareness and cognition, reciprocal social communication, social motivation and autistic behaviours. The results of this questionnaire are summed into raw and T-scores based on population norms [45]. Total T-scores of 76 and higher indicate severe problems and are associated with a clinical diagnosis of ASD. Total T-scores of between 60 and 75 are considered as being in the mild to moderate range, indicating deficiencies in reciprocal social interaction, which may be clinically significant. T-scores below 59 are considered as being in the normal range.

Attention deficit hyperactivity disorder (ADHD) was assessed in 17 children and adolescents with *NF1* microdeletions by means of test methods described in our previous study [18].

### Scoliosis

The presence of scoliosis was determined in 30 children with *NF1* microdeletions by X-ray analysis. A Cobb angle higher than 10 degree was considered as indicative of scoliosis.

### Statistical analysis

The number of patients with *NF1* microdeletions with or without externally visible or internal neurofibromas was compared with the number of patients from the general NF1 population or patients with intragenic *NF1* mutations of the control cohort using the Fisher’s exact test (https://www.langsrud.com/stat/Fishertest.htm). The median total volume of internal tumours in millilitres was compared between deletion and non-deletion patients using a non-parametric Mann-Whitney *U* test (https://www.socscistatistics.com/tests/mannwhitney/default2.aspx.) Mean values were compared using an unpaired *t* test (https://www.graphpad.com/quickcalcs/ttest2/). A difference with *p* < 0.05 was considered as significant. All statistical tests performed were two-sided.

## Results and discussion

In this study, we characterized the clinical phenotype of 30 children and adolescents with *NF1* microdeletions. The type and the extent of the *NF1* deletions were analysed in our previous studies or in the course of the current study as summarized in Supp. Table S1. Of these 30 patients, 27 harboured type-1 *NF1* deletions spanning 1.4 Mb, whereas three patients had larger atypical *NF1* deletions encompassing 2 Mb, 3 Mb and 4.7 Mb, respectively (Supp. Table S1).

### Cutaneous and subcutaneous neurofibromas

Cutaneous neurofibromas (cnf) and subcutaneous neurofibromas (scnf) are age-related clinical features of NF1. In the general NF1 population, these benign tumours are often not present in very young children but may appear around the age of 8 years, and their numbers increase with age [46]. Puberty and pregnancy often represent periods of neurofibroma growth [reviewed by 47, 48]. In children with NF1 younger than 10 years of age, only 3% present with cnf [49] or 11% according to Huson et al. [50]. By contrast, many children with *NF1* microdeletions seem to exhibit cnf and also scnf already at an early age [5, 9, 17, 29, 51, 52]. However, these studies included only a small number of young children, and the frequency of neurofibromas in children with *NF1* microdeletions has not been systematically analysed in a larger group of these patients. In the study presented here, we determined the number of cnf and scnf in 30 children and adolescents with *NF1* microdeletions (Supp. Table S2). We also assessed the absence or presence of these tumours in a control cohort of age-matched NF1 patients with intragenic *NF1* mutations analysed at the University Medical Center Hamburg-Eppendorf (Supp. Table S3). The comparison of both patient groups indicated that children and adolescents with *NF1* microdeletions exhibited significantly more often cnf and scnf than age-matched patients with intragenic *NF1* mutations (Table 1). Importantly, none of the patients investigated by us had several hundreds of cnf or scnf at an early age. Such high numbers of neurofibromas are typically seen in a subgroup of adult patients with NF1. Nevertheless, seven of the 30 children and adolescents with *NF1* microdeletions investigated by us had 50 or more cnf or scnf at the age of 18 years or younger (Supp. Table S2). In 13 children with *NF1* microdeletions and neurofibromas present at an early age, the number of tumours was assessed also at later years of their life. A considerable increase in the number of tumours with advancing age was observed in 8 (61%) of these 13 patients (Supp. Table S2). This finding implies that if neurofibromas are present at an early age in patients with *NF1* microdeletions, a further increase in the number of neurofibromas during childhood and adolescence is very likely.

**Table 1:** Comparison of the number of children and adolescents with neurofibromas (nf). The comparison included two groups of age-matched patients, those with *NF1* microdeletions and those with intragenic *NF1* mutations analysed at the University Medical left Hamburg Eppendorf, Germany

	Number of children with intragenic *NF1* mutations	Number of children with *NF1* microdeletions	*p* value^a^
Number of cutaneous nf			
0	24 (80%)	12 (40%)	0.003
≥ 1	6 (20%)	18 (60%)	
Number of subcutaneous nf			
0	28 (93%)	15 (50%)	0.0003
≥ 1	2 (7%)	15 (50%)	
Number of plexiform nf			
0	23 (77%)	14 (47%)	0.032
≥ 1	7 (23%)	16 (53%)	

^a^Two-tailed Fisher’s exact test

We also compared the number of children and adolescents with *NF1* microdeletions exhibiting cnf or scnf with those observed in the cohort of NF1 patients reported by Duong et al. [49]. The patients of this cohort were not selected pertaining to *NF1* mutation type and thus represent the general NF1 population. The analysis of this control cohort demonstrated that only 3% of the children younger 10 years of age had cnf, whereas 10% exhibited scnf [49]. By contrast, 54% of the *NF1* microdeletion patients younger than 10 years of age had cnf, and 41% exhibited scnf (Table 2).

**Table 2:** Comparison of the number of children with neurofibromas (nf). The comparison included two groups of patients, those with *NF1* microdeletions analysed in the present study and the NF1 children of the cohort reported by Duong et al. [49]. The patients analysed by Duong et al. were not selected pertaining to *NF1* mutation type and hence represent the general NF1 population. The children analysed were younger than 10 years

	Number of children analysed by Duong et al. [49]	Number of children with *NF1* microdeletions analysed in this study	*p* value^a^
Number of cutaneous nf			
0–1	66 (97%)	12 (46%)	4.9 × 10^−8^
≥ 2	2 (3%)	14 (54%)	
Number of subcutaneous nf			
0–1	61 (90%)	16 (59%)	0.0013
≥2	7 (10%)	11 (41%)	

^a^Two-tailed Fisher’s exact test

In children and adolescents with *NF1* microdeletions aged ≥ 10–19 years, 90% had cnf, and 67% exhibited scnf. By contrast, only 23% of the NF1 patients aged ≥ 10–19 years reported by Duong et al. [49] had cnf, and 26% of the patients exhibited scnf (Table 3). Taken together, children and adolescents with *NF1* microdeletions had significantly more often cnf and scnf than patients of the general NF1 population (Tables 2 and 3). This difference remained significant if the three patients with atypical *NF1* deletions larger than 1.4 Mb were not included in the comparison and only patients with type-1 *NF1* microdeletions were analysed (Supp. Tables S4 and S5).

**Table 3:** Comparison of the number of children and adolescents with neurofibromas (nf). The comparison included two groups of age-matched patients, those with *NF1* microdeletions analysed in the present study and the NF1 patients of the cohort reported by Duong et al. [49]. The patients analysed by Duong et al. were not selected pertaining to *NF1* mutation type and hence represent the general NF1 population. The patients analysed were ≥ 10–19 years of age

	Number of patients analysed by Duong et al. [49]	Number of patients with *NF1* microdeletions analysed in this study	*p* value^a^
Number of cutaneous nf			
0–1	131 (77%)	2 (10%)	6.6 × 10^−9^
≥ 2	40 (23%)	18 (90%)	
Number of subcutaneous nf			
0–1	127 (74%)	7 (33%)	0.0002
≥ 2	44 (26%)	14 (67%)	

^a^Two-tailed Fisher’s exact test

Also Huson et al. [50] investigated the proportion of children and adolescents with NF1 who exhibit cutaneous neurofibromas (cnf). The patients of this cohort were not selected with regard to *NF1* mutation type and thus represent the general NF1 population. A comparison between the patients of this cohort and the children and adolescents with *NF1* microdeletions analysed here indicated that children and adolescents with *NF1* microdeletions had significantly more often cnf than age-matched patients of the general NF1 population (Supp. Tables S6 and S7).

It seems likely to assume that the hemizygous loss of a gene or genes located within the 1.4-Mb microdeletion region promotes early neurofibroma growth in *NF1* microdeletion patients. The *MIR193A* gene is a good candidate for such a gene as it encodes for the microRNA miR-193a which represses c-kit expression [53, 54]. The tumour microenvironment, in particular c-kit expressing mast cells, has been shown to be important for neurofibroma growth in various mouse models [reviewed by 55]. Cutaneous neurofibromas are benign tumours that do not transform towards malignancy, and local inflammation mediated by activated mast cells plays a key role in the promotion of neurofibroma cell growth [reviewed by 55, 56, 57]. Mouse models indicated that the elimination of mast cells from the tumour microenvironment abrogated neurofibroma growth and that *Nf1* heterozygous mast cells require c-kit for tumour formation and maintenance [58]. Hence, the reduced miR-193a expression in patients with *NF1* microdeletions due to the hemizygous loss of the *MIR193A* gene may lead to enhanced c-kit expression and activation of mast cells which in turn contribute to early neurofibroma growth.

### Externally visible plexiform neurofibromas

Previously performed studies reported the frequency of externally visible plexiform neurofibromas in patients with NF1 of all age groups to be 32% [50], 15% [59] and 16.8% [60]. Plexiform neurofibromas (pnf) are considered to be tumours of early embryonic origin, but not all pnf are readily detected in very young children if the tumours are of small size, even although the growth rate of pnf is highest in young children [60–63]. In the study presented here, we investigated the presence or absence of externally visible pnf in children and adolescents with *NF1* microdeletions as well as age-matched patients with intragenic *NF1* mutations. Pnf were observed in 16 (53%) of the 30 *NF1* microdeletion patients. By contrast, only 7 (23%) of the 30 age-matched patients with intragenic *NF1* mutations exhibited pnf (*p* = 0.032; two-tailed Fisher’s exact test; Table 1, Supp. Table S3). If only children in the age group of 1–3 years were analysed, 8 (67%) of 12 children with *NF1* microdeletions had pnf. In contrast to this, only one child (9%) of the 11 children with intragenic *NF1* mutations at the age of 1–3 years had a plexiform neurofibroma (Supp. Table S8). This difference in frequency of pnf among very young children at the age of 1–3 years was significant (*p* = 0.0094; two-tailed Fisher’s exact test) and suggests that pnf tend to be larger and therefore earlier to identify in children with *NF1* microdeletions as compared with those with intragenic *NF1* mutations.

In our previous study, pnf were observed in 22 (76%) of 29 *NF1* microdeletion patients analysed [18]. These 29 patients were of different age (range: 4–43 years), and six of the young children analysed here were included in this previous study. If only the 19 adult *NF1* microdeletion patients from our previous study are considered (mean age 29.5 years, SD 9.6), pnf were present in 15 (79%) of them. This proportion is much higher than the 15–32% of patients from the general NF1 population who exhibit pnf [50, 59, 60]. Hence, our present study including children and adolescents with *NF1* microdeletions and our previous study on adult patients clearly indicate a very high frequency of pnf in patients with *NF1* microdeletions.

### Internal neurofibromas and MPNST

Whole-body MRI of NF1 patients indicates the burden of internal neurofibromas, most of which are not apparent on physical examination. At least 40% of adults with NF1 have internal neurofibromas as determined by whole-body MRI, whereas most of these tumours are asymptomatic [38, 64, 65].

In order to assess the internal tumour load in children and adolescents with *NF1* microdeletions, we performed whole-body MRI and volumetric analysis of all internal tumours identified (Supp. Table S2). This was possible for 20 *NF1* microdeletion patients investigated in this study, and we compared these results with the internal tumour load of 28 age-matched NF1 patients without large deletions analysed by us in a previous study [39]. Internal tumours were observed in all 20 *NF1* microdeletion patients analysed. By contrast, only 17 (61%) of the 28 non-deletion patients had internal tumours (Table 4). Thus, children and adolescents with *NF1* microdeletions had more frequently internal neurofibromas than NF1 patients without microdeletions (*p* = 0.0011; Table 4).

**Table 4:** Number of patients with and without *NF1* microdeletions exhibiting internal tumours as determined by whole-body MRI. The total internal tumour volume in millilitres (ml) was determined by volumetric analysis of MRI scans

	NF1 patients without *NF1* microdeletions	Patients with *NF1* microdeletions	*p* value
Number of patients analysed	28	20	
Number of patients with internal tumours	17/28 (61%)	20/20 (100%)	**0.0011**^**a**^
Mean age of all patients analysed (SD; range)	12.4 (4.9; 3–19 years)	8.7 (5.3; 1–19 years)	
Mean age of patients with internal tumours (SD; range)	12.5 (4.9; 3–18 years)	8.7 (5.3; 1–19 years)	
Total internal tumour volume in all patients:			
Median (range) [number of patients analysed]	5.5 ml (0–880) [*N* = 28]	140 ml (25–2500) [*N* = 20]	**0.0002**^**b**^
Mean (SD; 95% CI) [number of patients analysed]	112.6 ml (220; 31.1–194) [*N* = 28]	555.7 ml (768.0; 219–893) [*N* = 20]	**0.0057**^**c**^
Total internal tumour volume in patients with internal tumours:			
Median (range) [number of patients analysed]	41 ml (3–880) [*N* = 17]	140 ml (25–2500) [*N* = 20]	**0.0423**^**b**^
Mean (SD; 95% CI) [number of patients analysed]	185.5 ml (257.5; 63.5–308) [*N* = 17]	555.7 ml (768.0; 219–893) [*N* = 20]	**0.0216**^**c**^
Number of patients with total internal tumour volume ≥ 800 ml	1/17	6/20	0.097^**a**^

*p* values in bold indicate significance

^a^Two-tailed Fisher’s exact test

^b^Two-sided Mann-Whitney *U* test

^c^Two-sided unpaired *t* test

We also determined the total internal tumour load in millilitres (ml) by volumetric analysis in 20 children and adolescents with *NF1* microdeletions. The median total internal tumour volume was significantly higher in *NF1* microdeletion patients than in age-matched NF1 patients without deletions (*p* = 0.0002; Table 4). If only non-deletion patients with internal tumours were included in the comparison, the difference remained statistically significant (*p* = 0.0423; Table 4).

Also the mean total tumour volume was significantly higher in patients with *NF1* microdeletions than in NF1 patients without microdeletions (*p* = 0.0057; Table 4), if non-deletion patients without internal tumours were included in the analysis. Considering only patients with internal tumours, the mean total tumour volume remained to be significantly different comparing patients with and without *NF1* microdeletions (*p* = 0.0216; Table 4). Considerable differences in total internal tumour volume per patient were observed in both groups of patients (Supp. Table S9).

The frequency of a very high internal tumour burden, as arbitrarily defined by total tumour volume of ≥ 800 ml, was not significantly different between children and adolescents with and without *NF1* microdeletions (Table 4). Previously, we observed that an extremely high total internal tumour volume of > 3000 ml was significantly more frequent in patients with *NF1* microdeletions than in NF1 patients without large deletions [39]. However, such an extremely high volume of internal tumours is likely to be age-related, and our previous study included many adult patients. Of the 38 *NF1* deletion patients analysed in this previous study, 23 (61%) were adults. The mean age of these 38 patients was 26.1 years (SD 14.5, 95% CI 21.5–30.6; range: 3–69 years) [39]. The children and adolescents with large *NF1* deletions analysed in the present study had a mean age of 8.7 years (SD 5.3; 95% CI 6.24–11.1; range: 1–19 years). We conclude that an extremely high total internal tumour volume of > 3000 ml is significantly more frequent in adults with *NF1* microdeletions than in adults without large *NF1* deletions. Even although an extremely high internal tumour volume of > 3000 ml was not observed in the children and adolescents analysed here, it is noteworthy that two of the children with type-1 *NF1* microdeletions investigated by us exhibited a high internal tumour volume at a young age: female patient 450 had a total internal tumour volume of 1850 ml at the age of 9 years, whereas another girl, patient 1333, exhibited a total internal tumour volume of 2400 ml at the age of 12 years (Supp. Table S2). Equally high total internal tumour volumes were not observed in the group of patients without *NF1* microdeletions (Supp. Table S9).

Previous studies indicated that a high burden of internal neurofibromas is a risk factor for malignant peripheral nerve sheath tumour (MPNST) in NF1 [38, 66, 67]. Most MPNSTs in NF1 patients develop from pre-existing externally visible or deep-seated, internal plexiform neurofibromas [68, 69]. Thus, the frequent occurrence of internal tumours in children and adolescents with *NF1* microdeletions is important pertaining to their increased MPNST risk. Patients with *NF1* microdeletions have a lifetime MPNST risk in the range of 16–26% which is at least twice as high as the risk of patients with intragenic *NF1* mutations [18, 24]. Furthermore, MPNSTs seem to occur significantly earlier in patients with *NF1* microdeletions as compared with patients with intragenic *NF1* mutations [24]. De Raedt et al. [24] observed a substantial difference in the median age at diagnosis between patients with *NF1* microdeletion (22 years) and non-deletion cases (30 years). In the study of De Raedt et al. [24], nine patients with large *NF1* deletions and MPNSTs were investigated, and the youngest of these patients were 15, 17 and 18 years old.

In the group of patients with *NF1* microdeletion investigated by us, 20 were clinically analysed since early childhood and also later in life. These 20 patients were 13–32 years old at the last time of investigation, with a median age of 25 years. Three (15%) of them died because of an MPNST at the age of 13, 26 and 27 years, respectively. All three patients had a high load of internal neurofibromas as concluded from a total internal tumour volume of 1430 ml, 2400 ml and 2500 ml, respectively (Supp. Table S2). An internal tumour load of 2400 ml was observed in a girl with a type-1 *NF1* deletion who died at the age of 13 years because of an MPNST.

The increased risk of MPNSTs in patients with *NF1* microdeletions is associated with hemizygosity of the *SUZ12* gene, located within the *NF1* microdeletion region. Both *SUZ12* alleles are frequently inactivated in MPNSTs indicating its tumour suppressor function in this tumour type [70–72]. Our findings are in accordance with the view that in addition to *SUZ12* loss, a high load of internal neurofibromas is causally associated with the increased MPNST risk in patients with *NF1* microdeletions who need close monitoring by clinical examination and whole-body MRI performed already at an early age.

### Gliomas

The most common central nervous system tumours in NF1 are gliomas [reviewed by 73]. Gliomas usually affect children with NF1; the mean age at diagnosis is 4.5 years. The most common glioma associated with NF1 is pilocytic astrocytoma, a WHO grade I tumour, with the optic pathway (OP) glioma being the hallmark lesion [reviewed by 73]. An estimated 15% of all children with NF1 investigated by MRI have OP gliomas [74]. In our study, three (10 %) of the 29 children and adolescents with *NF1* microdeletions investigated by head MRI had non-symptomatic OP gliomas (Supp. Table S2). In the other 26 patients of our study group, neither symptomatic nor non-symptomatic OP gliomas were observed by MRI. Our findings do not indicate an increased OP glioma risk in children with *NF1* microdeletions as compared with the general NF1 population (Supp. Table S10).

Non-OP gliomas, located anatomically distinct from the optic pathway, occur in 33–64% of children with NF1 [75–77]. In only one of the 29 *NF1* microdeletion patients investigated by means of brain MRI, a non-OP glioma located in the brainstem was diagnosed at the age of 18 years (Supp. Table S2). Our findings suggest that also non-OP gliomas are not more frequent in children and adolescents with *NF1* microdeletions than in the general NF1 population.

### Skeletal anomalies

Scoliosis was observed in 16 (53%) of the 30 children with *NF1* microdeletions investigated by us (Supp. Table S11). In previously performed studies including mainly adults with *NF1* microdeletions, 43% exhibited scoliosis [17, 18]. The frequency of scoliosis in the general NF1 population is approximately 25% [78]. Our findings confirm that scoliosis is significantly more frequent in patients with *NF1* microdeletions than in the general NF1 population (*p* = 0.0026, two-tailed Fisher’s exact test).

Pes cavus (hollow foot) was observed in 6 (21%) of 29 children with *NF1* microdeletions analysed pertaining to this feature (Supp. Table S11). The frequency of this deformity in all patients with NF1 has not assessed as yet. However, pes cavus is not rare in the general population without NF1. In children of the general population at the age of 4–10 years, the frequency of pes cavus is 7%, and in the age group of 10–20 years, the frequency is 14% [79].

Pectus excavatum (PE) presents as a depression in the anterior chest wall resulting of a deviation of the dorsal sternum and the third to seventh rib or costal cartilage. The prevalence of PE in the normal population is 1:300–1000 [80, 81]. The frequency of pectus excavatum in the general NF1 population has been estimated to be up to 30% [82]. By contrast, Miraglia et al. [83] observed PE in only 12 (1%) of 1157 NF1 patients not selected pertaining to *NF1* mutation type. We observed PE in 6 (21%) of the 29 children with *NF1* microdeletions analysed here (Supp. Table S11). Further studies are necessary to investigate whether PE occurs more frequently in patients with *NF1* microdeletions than in patients with intragenic *NF1* mutations.

### Heart defects

Echocardiography was performed in 18 of the children with *NF1* microdeletions investigated here, and heart defects were observed in 9 (50%) of them. The heart defects included ventricular septal defect, pulmonic stenosis, atrial septal defect, aortic insufficiency and hypertrophic cardiomyopathy (Supp. Table S11). In a previous study, we observed cardiac defects in 6 (38%) of 16 *NF1* microdeletion patients but none in 16 age-matched NF1 patients with intragenic *NF1* mutations [20]. Hence, our present study confirms that congenital heart defects are frequent among patients with *NF1* microdeletion and may cause severe clinical complications.

### Facial dysmorphic features

Dysmorphic facial features including hypertelorism, broad fleshy nose, coarse facial appearance, flat forehead, deep front hair line, broad neck and low-set ears were observed in many of the *NF1* microdeletion patients investigated (Supp. Figure S1). However, in particular in small children, some of these dysmorphic facial features were mild or even missing. In these cases, the facial appearance would not have immediately raised the suspicion of the presence of a large *NF1* deletion.

### Developmental delay

Infants with NF1 frequently have developmental difficulties across different cognitive and motor domains, and these delays are often apparent already at the end of the first year of life [84, reviewed by 85, 86–88]. It has been estimated that 68% of all children with NF1 exhibit delays in at least one of various areas of motor and/or language skills [86]. Longitudinal analysis of developmental delays in children with NF1 which were not preselected with regard to *NF1* mutation type indicated that in particular motor delays do not improve over time [89]. Previous studies suggested that global developmental delay even more severe than the delay observed in many children from the general NF1 population is frequent in children with *NF1* microdeletions [reviewed by 14]. However, this has not been systematically assessed as yet in a larger number of patients. In the present study, we observed global developmental delay in motor skills and speech in 28 (93%) of the 30 children with *NF1* microdeletions investigated by us (Supp. Table S12). These delays were already apparent in young children (1–3 years of age), and the severe delays persisted during later childhood. Our study implies that severe global developmental delay is very frequent in children with *NF1* microdeletions. Further detailed studies including children of different age groups, analysed by well-suited and standardized evaluation methods, are necessary to characterize and quantify all areas affected by the developmental delay observed in children with *NF1* microdeletions in order to optimize the therapy for this specific group of NF1 patients who needs early intensive and long-term supportive intervention.

Five of the 30 children with *NF1* microdeletions investigated here were in the preschool age at the time of investigation. For 25 patients with *NF1* microdeletions, longitudinal assessments were available pertaining to their school performance and education. Nine (36%) of these 25 *NF1* microdeletion patients attended a school for children with learning disabilities or for children with physical and cognitive deficits, whereas another nine patients (36%) were students with special needs in inclusion education. Seven (28%) patients attended secondary school as regular pupils (Supp. Table S12). We conclude that despite of the global developmental delays observed in most patients with *NF1* microdeletions, there is variability pertaining to their performance at school as well as occupation later in life (Supp. Table S12).

### FSIQ

Children with NF1 often experience cognitive and behavioural problems [90, 91, reviewed by 85 and 92]. Many studies indicated that the mean full-scale IQ (FSIQ) of children with NF1 is around 90 with a standard deviation of 11–15 (23, 93, reviewed by 85]. Thus, the mean FSIQ in children with NF1 is lower than that of children from the general population. However, variance and heritability of IQ in individuals with NF1 are similar to that of the general population and mostly driven by genetic background differences [23]. Patients with *NF1* microdeletions exhibit significantly lower mean FSIQ scores than individuals carrying intragenic pathogenic *NF1* mutations [18, 22, 23]. In the study presented here, we determined FSIQ scores in 24 children and adolescents with *NF1* microdeletions investigated at the age of 6–18 years. The mean FSIQ in our patient group was 77.7 (SD 12.8) which is comparable with the findings of the previously performed studies as summarized in Table 5. Descheemaeker et al. [22] investigated 11 patients with *NF1* microdeletions who exhibited a mean FSIQ of 76 (SD 6.9). By contrast, the mean FSIQ was 88.5 in 106 children without *NF1* microdeletion investigated by these authors [22]. Taken together, previous studies and the results presented here indicate that the mean FSIQ in patients with *NF1* microdeletions is significantly lower than in patients with intragenic *NF1* mutations. However, as emphasized by Descheemaeker et al. [22], there is a substantial overlap between the FSIQ measures in NF1 patients with and without microdeletions. Furthermore, considerable heterogeneity of FSIQ scores is observed comparing individual patients with *NF1* microdeletions. In our present study, five (21%) of the 24 patients with *NF1* microdeletions analysed had an FSIQ below 70 indicative of mild intellectual disability. Only one of these five patients had an atypical 4.7-Mb spanning deletion, and the others exhibited the frequent type-1 *NF1* microdeletions of 1.4 Mb. Previously performed studies indicated an FSIQ < 70 in 18%, 35% and 41% of *NF1* microdeletions patients [18, 22, 23] (Table 5). Thus, intellectual disability is observed only in a subgroup of patients with *NF1* microdeletions.

**Table 5:** FSIQ in patients with *NF1* microdeletions analysed by Descheemaeker et al. [22], Mautner et al. [18], Ottenhoff et al. [23] and in the present study

	Descheemaeker et al. [22]	Mautner et al. [18]	Ottenhoff et al. [23]	This study
Total number of patients analysed	11	17	17	24
Mean FSIQ (SD; 95% CI)	76.0 (6.9; 71.4–80.6)	77.9 (14.3; 71.1–84.7)	71.2 (10.3; 66.3–76.1)	77.7 (12.8; 72.6–82.8)
Median FSIQ	79	75	71	76
FSIQ range	65–85	49–104	60–92	51–110
Number of patients with FSIQ < 70	2 (18%)	6 (35%)	7 (41%)	5 (21%)
Number of patients with FSIQ ≥ 70–< 85	8 (73%)	4 (24%)	8 (47%)	12 (50%)
Number of patients with FSIQ 85	1 (9%)	2 (12%)	0	1 (4%)
Number of patients with FSIQ > 85	0	5 (29%)^a^	2 (12%)^b^	6 (25%)^c^

All 11 patients analysed by Descheemaeker et al. and all 17 patients analysed by Mautner et al. had type-1 *NF1* deletions of 1.4 Mb. Among the 17 patients investigated by Ottenhoff et al., nine patients had type-1 *NF1* deletions, two deletions were atypical, one deletion was of type-2 and five deletions were not further characterized with regard to length. In our study, three of the 24 patients analysed had atypical *NF1* microdeletions of 4.7 Mb, 3 Mb and 2 Mb, respectively

The 17 patients with *NF1* microdeletions analysed by Ottenhoff et al. [23] indicated in this table do not include the patients analysed by Descheemaeker et al. [22]. Six patients included in our present study have been already analysed previously by Mautner et al. [18]. These six patients are not among the 17 *NF1* microdeletion patients analysed by Mautner et al. [18] included in this table

^a^The FSIQ values in these patients were 90, 91, 92, 99 and 104, respectively

^b^The FSIQ values in these patients were 88 and 92, respectively

^c^The FSIQ values in these patients were 88, 90, 91, 92, 97 and 110, respectively

In our study, 12 (50%) of the 24 patients with *NF1* microdeletions had an FSIQ ≥ 70–< 85. Remarkably, six of our patients with type-1 *NF1* deletions had an FSIQ higher than 85 (Table 5, Supp. Table S12). The heterogeneity pertaining to the cognitive capabilities in the group of patients with *NF1* microdeletions suggests that cognition in these patients is influenced by additional genetic and also environmental factors. Early therapeutic interventions to promote better achievements of functional potential later in life are very important in this group of patients.

Remarkably, one of our patients (patient 1261) had an FSIQ score of 110 and did not exhibit developmental delay. The patient has a type-1 *NF1* deletion of 1.4-Mb as determined by MLPA of blood leucocytes which did not indicate mosaicism with normal cells. However, MLPA is not sensitive enough to detect normal cells without the deletion present at proportions of less than 20%. Hence, we cannot exclude somatic mosaicism with normal cells not harbouring the *NF1* microdeletion in patient 1261 which would explain the high FSIQ and the absence of developmental delay in this patient. However, somatic mosaicism with normal cells not harbouring the *NF1* microdeletion is very rare in patients with type-1 *NF1* deletions [12, 13].

### ADHD

It has been estimated that up to 50% of all NF1 patients present with attention deficit hyperactivity disorder (ADHD) [reviewed by 94]. NF1 patients with ADHD score lower in intelligence tests than healthy (sibling) controls and exhibit lower IQ scores than NF1 patients without ADHD [90, 94–98]. In patients with NF1, attention deficits are a specific additional risk factor for suboptimal intellectual performance [99]. Irrespective of cognitive deficits, elevated ADHD symptoms in children with NF1 have been shown to impair considerably daily life functioning [98]. In the study presented here, ADHD was diagnosed in 15 (88%) of 17 children and adolescents with *NF1* microdeletion investigated (Supp. Table S12). We conclude that the high frequency of ADHD in patients with *NF1* microdeletions contributes considerably to the academic underachievement observed in this group of patients. Early interventions to minimize ADHD symptoms may help to reduce the functional impairment of children with *NF1* microdeletions.

### ASD

Autism spectrum disorder (ASD) has been estimated to affect 0.8% of the general population [100]. Previous studies demonstrated a high incidence of ASD and a substantially elevated autistic trait burden in individuals with NF1 [93, 101, 102, reviewed by 103]. Social problems in children with NF1 are more prevalent in those with low intellectual functioning [104].

ASD symptoms can be assessed by different means; one of the acknowledged assessment tools is the social responsiveness scale (SRS) questionnaire [44]. The result of this questionnaire is converted into a T-score which indicates the presence or absence and the severity of autistic symptomatology. Clinical ASD, as measured by a T-score ≥ 76, affects 8.7% or 13.2% of NF1 patients according to the study of Morris et al. [105] and Eijk et al. [93], respectively (Table 6). Subclinical autistic symptoms as measured by T-scores ≥ 60–75 have been observed in 16.5% of NF1 patients as reported by Eijk et al. [93] and in 26% of the NF1 patients investigated by Morris et al. [105]. The patients of both cohorts were not selected pertaining to *NF1* mutation type and hence are considered to represent the general NF1 population. Here, we determined T-scores in 24 patients with *NF1* microdeletions at the age of 4–18 years. T-scores ≥ 76 were observed in two (8.3%) of the patients with *NF1* microdeletions analysed in our study. Both patients were male, one with an atypical 4.7-Mb *NF1* deletion and the other with a type-1 *NF1* deletion of 1.4 Mb (Supp. Table S12). Consequently, severe, clinical ASD does not seem to be more frequent in patients with *NF1* microdeletions as compared with the general NF1 population. However, T-scores ≥ 60–75 were observed in 17 (70.8%) of the 24 *NF1* microdeletion patients analysed. Hence, T-scores ≥ 60–75, indicative of mild to moderate autistic symptoms, are significantly more frequent in patients with *NF1* microdeletions than in the general NF1 population (Table 6, Supp. Tables S12 and S13). Also the mean total T-score was significantly higher in patients with *NF1* microdeletions than in the general NF1 population (Table 6).

**Table 6:** Comparison of the frequency of autistic symptoms in 24 patients with *NF1* microdeletions analysed here and NF1 patients reported previously [93, 105]. The patients investigated by Eijk et al. [93] and Morris et al. [105] were not selected pertaining to *NF1* mutation type and thus represent the general NF1 population. Autistic symptoms were assessed by means of T-scores obtained by SRS questionnaires. The general population has a mean total T-score of 50 (SD 10)

	Morris et al. [105]	Eijk et al. [93]	Patients with *NF1* microdeletions analysed in this study	*p* value^a^	*p* value^b^
Total number of patients analysed	531	103	24		
T-score ≥ 76	70 (13.2%)	9 (8.7%)	2 (8.3%)		
T-score ≥ 60–75	138 (26.0%)	17 (16.5%)	17 (70.8%)	2.4 × 10^−7c^	1.1 × 10^−5c^
T-score < 60	323 (60.8%)	77 (74.8%)	5 (20.8%)		
Mean total T-score [SD]	58.2 [13.4]	54.7 [12.6]	64.6 [7.3]	0.0003^d^	0.0205^d^

^a^Eijk et al. [93] vs. this study

^b^Morris et al. [105] vs. this study

^c^Two-sided Fisher’s exact test

^d^Two-sided unpaired *t* test

*SD* standard deviation

Among the 24 patients with *NF1* microdeletions investigated by us were 15 males and nine females. Previous analyses indicated that male NF1 patients had significantly higher mean total T-scores relative to females [93, 101, 105]. A male predominance of ASD is also observed in the general population [106]. However, the mean T-scores in female and male patients with *NF1* microdeletions investigated by us were not significantly different (Supp. Table S14). Nevertheless, the low number of patients analysed by us may mask a putative male predominance among patients with *NF1* microdeletions and a high autistic trait burden. Taken together, 19 (79%) of the 24 *NF1* microdeletion patients investigated by us had T-scores ≥ 60 and thus exhibited autistic symptomatology. The high frequency of neuropsychiatric phenotypes such as autism and hyperactivity-inattention as well as cognitive deficits observed in patients with *NF1* microdeletions emphasizes the need for comprehensive social and therapeutic intervention to improve their executive function and academic performance.

## Conclusion

Our study indicates a prevalence of global developmental delay, cognitive deficits, attention deficit and autistic symptomatology in children with *NF1* microdeletions which is significantly higher than that observed in the general NF1 population. Our findings advocate an early screening and intensive support for children with *NF1* microdeletions who represent an at-risk paediatric subgroup of severely affected NF1 patients. Furthermore, our results indicate a high frequency of cutaneous and subcutaneous neurofibromas in children and adolescents with *NF1* microdeletions. Importantly, children and adolescents with *NF1* microdeletions exhibit more often externally visible and internal plexiform neurofibromas than age-matched patients with intragenic *NF1* mutations. The high prevalence of these tumours and their potential to transform towards malignancy as well as the loss of the *SUZ12* gene included in the *NF1* microdeletion region represent predisposing factors which increase the MPNST risk of patients with large *NF1* deletions. Children and adolescents with *NF1* microdeletions are a high-risk group for malignancy; whole-body MRI and long-term follow-up surveillance at a specialist NF1 network clinic are recommended.

## Electronic supplementary material

ESM 1 (PPTX 2624 kb)

ESM 2 (DOCX 58 kb)
