# Supplementary material for: Clinical characterization of children and adolescents with NF1 microdeletions
Source: Childs Nerv Syst. 2020 Jun 12;36(10):2297–310. doi: 10.1007/s00381-020-04717-0 (PMC7575500; doi:10.1007/s00381-020-04717-0)
Supplement: Supplementary file 1 — (PPTX 2624 kb) [file 381_2020_4717_MOESM1_ESM.pptx]

## Slide 1
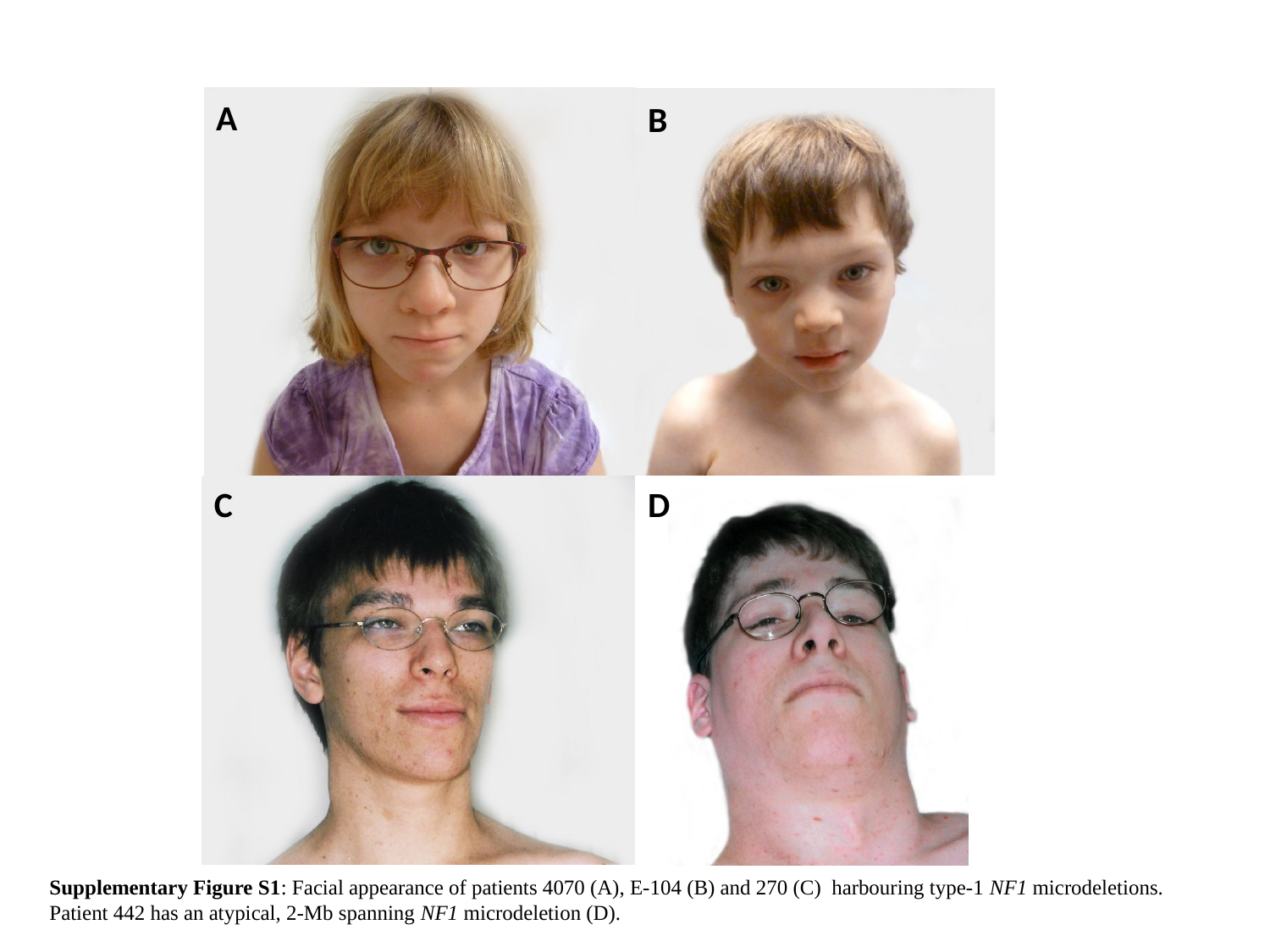

A
B
C
D
Supplementary Figure S1: Facial appearance of patients 4070 (A), E-104 (B) and 270 (C) harbouring type-1 NF1 microdeletions. Patient 442 has an atypical, 2-Mb spanning NF1 microdeletion (D).
