# Supplementary material for: Clinical characterization of children and adolescents with NF1 microdeletions
Source: Childs Nerv Syst. 2020 Jun 12;36(10):2297–310. doi: 10.1007/s00381-020-04717-0 (PMC7575500; doi:10.1007/s00381-020-04717-0)
Supplement: Supplementary file 2 — (DOCX 58 kb) [file 381_2020_4717_MOESM2_ESM.docx]

# Supplementary Table S1:

List of the patients with *NF1* microdeletions analysed in this study. The size and type of the deletion has been either characterized in our previous studies as indicated in the column entitled “reference” or newly determined during the course of the present study by MLPA (Kit P122, MRC Holland) or microarray analysis (CytoScan™ HD array, Affymetrix). The patients marked in bold letters have been clinically analysed by us in our previous study [18].

| **ID Number** | **Sex** | **Type of deletion (breakpoint localization)** | **Size of the deletion** | **Reference** |
| --- | --- | --- | --- | --- |
| E-102 | m | type-1 (not analyzed) | 1.4-Mb | this study, MLPA |
| 4904,0 | m | type-1 (not analyzed) | 1.4-Mb | this study, MLPA |
| 2483 | m | atypical | 4.7-Mb | Kehrer-Sawatzki et al. [31] |
| **1333** | f | type-1 (PRS2) | 1.4-Mb | Hillmer et al. [36] |
| 1598 | m | type-1 (PRS2) | 1.4-Mb | Hillmer et al. [36] |
| 4092 | f | type-1 (PRS2) | 1.4-Mb | Hillmer et al. [36] |
| 3415 | m | type-1 (PRS2) | 1.4-Mb | Summerer et al. [37] |
| E-103 | m | type-1 (not analyzed) | 1.4-Mb | this study, MLPA |
| E-101 | m | type-1 (not analyzed) | 1.4-Mb | this study^,^ microarray |
| 4631 | f | type-1 (not analyzed) | 1.4-Mb | this study, MLPA |
| **2490** | f | type-1 (PRS2) | 1.4-Mb | Hillmer et al. [36] |
| 3791 | m | type-1 (PRS2) | 1.4-Mb | Summerer et al. [37] |
| 4061 | m | type-1 (PRS1) | 1.4-Mb | Hillmer et al. [36] |
| 442 | m | atypical | 2-Mb | Kehrer-Sawatzki et al. [32] |
| 4253 | f | type-1 (PRS1) | 1.4-Mb | Hillmer et al. [35] |
| **450** | f | type-1 (PRS2) | 1.4-Mb | Hillmer et al. [35, 36] |
| 1261 | m | type-1 (not analyzed) |  | this study, MLPA |
| E-104 | m | type-1 (not analyzed) | 1.4-Mb | this study, microarray |
| **284** | f | type-1 (PRS2) | 1.4-Mb | Hillmer et al. [35] |
| 1807 | m | type-1 (PRS2) | 1.4-Mb | Hillmer et al. [36] |
| DN56837 | f | type-1 (not analyzed) | 1.4-Mb | this study, MLPA |
| 3350 | m | type-1 (PRS2) | 1.4-Mb | Hillmer et al. [36] |
| 4070 | f | type-1 (PRS2) | 1.4-Mb | Hillmer et al. [36] |
| PFA | m | type-1 (PRS2) | 1.4-Mb | Bengesser et al. [33]; Hillmer et al. [36] |
| 3751 | f | type-1 (PRS1) | 1.4-Mb | Hillmer et al. [35, 36] |
| 3772 | m | type-1 (PRS2) | 1.4-Mb | Summerer et al. [37] |
| **867** | f | type-1 (PRS2) | 1.4-Mb | Hillmer et al. [35] |
| E-105 | m | type-1 (PRS2) | 1.4-Mb | Summerer et al. [37] |
| 619/430 | f | atypical | 3-Mb | Vogt et al. [34] |
| **270** | m | type-1 (PRS1) | 1.4-Mb | Hillmer et al. [36] |

PRS1 (paralogous recombination site 1); PRS2 (paralogous recombination site 2)

# Supplementary Table S2:

Tumour load of the *NF1* microdeletion patients investigated in this study. The age of the patient at the time of investigation is indicated in square brackets. n.a.: not analaysed; y: year(s)

| **ID Number** | **Deletion size in Mb** | **Number of externally visible neurofibromas** | | | **Total internal tumour volume^a^** | **Brain**  **tumour^b^** | **MPNST (age of death)^c^** |
| --- | --- | --- | --- | --- | --- | --- | --- |
|  |  | **plexiform** | **cutaneous** | **subcutaneous** |  |  |  |
| E-102 | 1.4 | 1 [1 y] | 0 [1 y] | 1 [1 y] | 25 ml [1 y] | 0 [1 y] | 0 [2 y] |
| 4904,0 | 1.4 | 1 [2 y] | 0 [2 y] | 0 [2 y] | 90 ml [2 y] | 0 [2 y] | 0 [3 y] |
| 2483 | 4.7 | 0 [2 y]  0 [19 y] | 5-10 [3 y]  20-30 [4 y]  >1000 [19 y] | 0 [19 y] | 1430 ml [26 y] | 0 [27 y] | + (26 y) |
| 1333 | 1.4 | 3 [2 y] | 0 [10 y] | 20 [2 y]  **200 [10 y]** | 2400 ml [12 y] | 0 [12 y] | + (13 y) |
| 1598 | 1.4 | 1 [2 y] | 0 [2 y]  **50 [12 y]** | 2 [2 y]  **50 [2 y]** | 100 ml [15 y] | 0 [15 y] | 0 [15 y] |
| 4092 | 1.4 | 0 [2 y] | 0 [2 y] | 10 [2 y] | 400 ml [10 y] | 0 [10 y] | 0 [15 y] |
| 3415 | 1.4 | 0 [2 y] | 0 [2 y]  0 [7 y] | 8 [7 y] | 100 ml [4 y] | 0 [4 y] | 0 [12 y] |
| E-103 | 1.4 | 1 [2 y] | 2 [3 y] | 0 [3 y] | 50 ml [2 y] | asymptomatic optic pathway glioma [2 y] | 0 [3 y] |
| E-101 | 1.4 | 1 [3 y] | 0 [3 y] | 5 [3 y] | 801 ml [3 y] | 0 [3 y] | 0 [5 y] |
| 4631 | 1.4 | 0 [3 y] | 0 [3 y]  20 [25 y] | 0 [3 y]  0 [25 y] | 100 ml [3 y] | 0 [3 y] | 0 [27 y] |
| 2490 | 1.4 | 1 [3 y]  3 [6 y] | 10 [3 y]  15 [6 y]  25 [10 y]  **70 [12 y]** | 15 [6 y]  25 [10 y] | 200 ml [4 y] | asymptomatic optic pathway glioma [4 y] | 0 [15 y] |
| 3791 | 1.4 | 1 [3 y] | 6 [3 y] | 0 [3 y] | n.a. | 0 [3 y] | 0 [28 y] |
| 4061 | 1.4 | 0 [4 y] | 10 [4 y] | 0 [4 y] | n.a. | n.a. | 0 [10 y] |
| 442 | 2 | 1 [4 y] | 0 [4 y] | 15 [4 y]  **700 [18 y]**  >1000 [22 y] | n.a | 0 [37 y] | 0 [37 y] |
| 4253 | 1.4 | 3 [4 y] | 5 [4 y] | 5 [4 y] | n.a. | 0 [4 y] | 0 [9 y] |
| 450 | 1.4 | 0 [4 y] | 6 [4 y]  20 [12 y] | 0 [12 y] | 1850 ml [9 y] | 0 [9 y] | 0 [25 y] |
| 1261 | 1.4 | 0 [5 y] | 2 [5 y]  4 [7 y] | 0 [5 y] | 57 ml [7 y] | 0 [7 y] | 0 [19 y] |
| E-104 | 1.4 | 3 [5 y] | 15 [5 y] | 0 [5 y] | n.a. | 0 [5 y] | 0 [5 y] |
| 284 | 1.4 | 4 [6 y] | 3 [6 y] | 20 [13 y] | n.a. | 0 [7 y] | 0 [29 y] |
| 1807 | 1.4 | 0 [7 y] | 5 [7 y] | 5 [7 y]  20-30 [21 y] | 180 ml [13 y] | 0 [16 y] | 0 [25 y] |
| DN56837 | 1.4 | 0 [8 y] | 0 [8 y] | 0 [8 y] | n.a. | 0 [8 y] | 0 [10 y] |
| 3350 | 1.4 | 2 [8 y] | 20 [8 y] | 0 [8 y] | 20 ml [11 y] | 0 [11 y] | 0 [14 y] |
| 4070 | 1.4 | 0 [2 y] | 0 [9 y] | 10 [9 y] | 50 ml [7 y] | 0 [7 y] | 0 [15 y] |
| PFA | 1.4 | 0 [9 y] | 11 [9 y] | **>100 [9 y]**  >1000 [24 y] | n.a. | brainstem glioma [18 y] | 0 [28 y] |
| 3751 | 1.4 | 0 [9 y] | 6 [9 y]  30 [10 y]  **100 [13 y]** | 0 [13 y] | 400 ml [10 y] | 0 [10 y] | 0 [16 y] |
| 3772 | 1.4 | 2 [11 y] | 20 [11 y]  25 [15 y] | ~30 [15 y] | 900 ml [19 y] | 0 [20 y] | 0 [26 y] |
| 867 | 1.4 | 1 [12 y] | 0 [12 y] | 5 [10 y]  10 [12 y] | 90 ml [9 y] | asymptomatic optic pathway glioma [9 y] | 0 [30] |
| E-105 | 1.4 | 0 [14 y] | **70 [14 y]**  >1000 [22 y] | 0 [22 y] | 800 ml [14 y] | 0 [14 y] | 0 [23 y] |
| 619/430 | 3 | 0 [15 y] | 20 [15 y]  30 [20 y] | 0[15 y] | n.a. | n.a. | 0 [32 y] |
| 270 | 1.4 | 3 [15 y] | 20-30 [16 y] | 0 [16 y] | 2500 ml [18 y] | 0 [18 y] | + (27 y) |

a: the volume of all internal tumours was determined by whole-body MRI and volumetric analysis. The load of internal neurofibromas is given as total tumour volume in milliliters (ml).

b: as determined by MRI.

c: age of death because of the MPNST.

Marked in bold are cases with >50 neurofibromas at the age of 18 years or younger

# Supplementary Table S3:

Comparison between patients with intragenic *NF1* mutations and patients with large *NF1* deletions pertaining to the presence (+) or absence (0) of plexiform neurofibromas (pnf), cutaneous neurofibromas (cnf) and subcutaneous neurofibromas (scnf). Indicated is the age at investigation in years (y).

| Patients with intragenic *NF1* mutations | | | | | Patients with *NF1* microdeletions | | | | |
| --- | --- | --- | --- | --- | --- | --- | --- | --- | --- |
| ID | age (y) | pnf | cnf | scnf | ID | age (y) | pnf | cnf | scnf |
| 212 | 1 | 0 | 0 | 0 | E-102 | 1 | + | 0 | + |
| 22 | 2 | 0 | 0 | 0 | 4904,0 | 2 | + | 0 | 0 |
| 118 | 3 | 0 | 0 | 0 | 2483 | 3 | 0 | + | 0 |
| 515 | 2 | 0 | 0 | 0 | 1333 | 2 | + | 0 | + |
| 211 | 2 | 0 | 0 | 0 | 1598 | 2 | + | 0 | + |
| 420 | 1 | 0 | 0 | 0 | 4092 | 2 | 0 | 0 | + |
| 213 | 1 | 0 | 0 | 0 | 3415 | 2 | 0 | 0 | + |
| 410 | 2 | 0 | 0 | 0 | E-103 | 2 | + | + | 0 |
| 210 | 1 | + | 0 | 0 | E-101 | 3 | + | 0 | + |
| 114 | 2 | + | 0 | 0 | 4631 | 3 | 0 | 0 | 0 |
| 11 | 1 | 0 | 0 | 0 | 2490 | 3 | + | + | + |
| 210 | 5 | 0 | 0 | 0 | 3791 | 3 | + | + | 0 |
| 1113 | 4 | 0 | 0 | 0 | 4061 | 4 | 0 | + | 0 |
| 412 | 4 | 0 | 0 | 0 | 442 | 4 | + | 0 | + |
| 612 | 5 | 0 | + | + | 4253 | 4 | + | + | + |
| 714 | 5 | 0 | 0 | 0 | 450 | 4 | 0 | + | 0 |
| 313 | 5 | 0 | 0 | 0 | 1261 | 5 | 0 | + | 0 |
| 65 | 5 | 0 | + | + | E-104 | 5 | + | + | 0 |
| 42 | 6 | 0 | 0 | 0 | 284 | 6 | + | + | + |
| 2310 | 7 | + | 0 | 0 | 1807 | 7 | 0 | + | + |
| 812 | 8 | 0 | 0 | 0 | DN56837 | 8 | 0 | 0 | 0 |
| 420 | 7 | + | 0 | 0 | 3350 | 8 | + | + | 0 |
| 1112 | 8 | 0 | 0 | 0 | 4070 | 9 | 0 | 0 | + |
| 1114 | 9 | 0 | + | 0 | PFA | 9 | 0 | + | + |
| 810 | 8 | + | 0 | 0 | 3751 | 9 | 0 | + | 0 |
| 1210 | 11 | 0 | 0 | 0 | 3772 | 11 | + | + | + |
| 1112 | 12 | + | 0 | 0 | 867 | 12 | + | 0 | + |
| 1914 | 14 | + | + | 0 | E-105 | 14 | 0 | + | 0 |
| 112 | 14 | 0 | + | 0 | 619/430 | 14 | 0 | + | 0 |
| 1820 | 15 | 0 | + | 0 | 270 | 15 | + | + | 0 |

# Supplementary Table S4:

Comparison of the number of children and adolescents with neurofibromas (nf). The comparison included two groups of age-matched patients, those with *NF1* microdeletions analysed in the present study and the NF1 children of the cohort reported by Duong et al. [49]. The patients analysed by Duong et al. were not selected pertaining to *NF1* mutation type and hence represent the general NF1 population. The children analysed were younger than 10 years of age. Patients with atypical *NF1* deletions larger than 1.4-Mb were not included in this comparison.

|  | Number of children analysed by Duong et al. [49] | Number of children with *NF1* microdeletions analysed here | P-value ^a^ |
| --- | --- | --- | --- |
| Number of cutaneous nf |  |  |  |
| 0 - 1 | 66 | 11 | 8.8 x 10^-8^ |
| ≥ 2 | 2 (3%) | 13 (54%) |  |
| Number of subcutaneous nf |  |  |  |
| 0 - 1 | 61 | 14 | 0.0015 |
| ≥ 2 | 7 (11%) | 10 (42%) |  |

a: two-tailed Fisher’s exact test

# Supplementary Table S5:

Comparison of the number of children and adolescents with neurofibromas (nf). The comparison included two groups of age-matched patients, those with *NF1* microdeletions analysed in the present study and the NF1 children of the cohort reported by Duong et al. [49]. The patients analysed by Duong et al. were not selected pertaining to *NF1* mutation type and hence represent the general NF1 population. The patients analysed were ≥ 10-19 years of age. Patients with atypical *NF*1 deletions larger than 1.4-Mb were not included in this analysis.

|  | Number of patients analysed by Duong et al. [49] | Number of patients with *NF1* microdeletions analysed here | P-value ^a^ |
| --- | --- | --- | --- |
| Number of cutaneous nf |  |  |  |
| 0 - 1 | 131 | 2 | 5.9 x 10^-8^ |
| ≥ 2 | 40 (23%) | 16 (89%) |  |
| Number of subcutaneous nf |  |  |  |
| 0 - 1 | 127 | 5 | 0.0001 |
| ≥ 2 | 44 (26%) | 13 (72%) |  |

a: two-tailed Fisher’s exact test

# Supplementary Table S6:

Comparison of the number of children and adolescents with neurofibromas (nf). The comparison included two groups of age-matched patients, those with *NF1* microdeletions analysed in the present study and the NF1 children of the cohort reported by Huson et al. [50]. The patients analysed by Huson et al. [50] were not selected pertaining to *NF1* mutation type and hence represent the general NF1 population. The children analysed were younger than 10 years of age.

|  | Number of children analysed by Huson et al. [50] | Number of children with *NF1* microdeletions (type-1 deletions only) | P-value ^a^ |
| --- | --- | --- | --- |
| Number of cutaneous nf |  |  |  |
| 0 | 15 | 12 (11) | 0.009  (0.008) |
| ≥ 1 | 2 | 14 (13) |  |

a: two-tailed Fisher’s exact test. The P-value in parenthesis was calculated for the comparison between the number of patients with cutaneous neurofibromas and type-1 *NF1* deletions of 1.4-Mb and the number of patients with cutaneous neurofibromas from the control cohort. Patients with atypical *NF1* deletions larger than 1.4-Mb were not included in this comparison.

# Supplementary Table S7:

Comparison of the number of children and adolescents with neurofibromas (nf). The comparison included two groups of age-matched patients, those with *NF1* microdeletions analysed in the present study and the NF1 children of the cohort reported by Huson et al. [50]. The patients analysed by Huson et al. [50] were not selected pertaining to *NF1* mutation type and hence represent the general NF1 population. The patients analysed were ≥ 10-19 years of age.

|  | Number of patients analysed by Huson et al. [50] | Number of patients with *NF1* microdeletions (type-1 deletions only) | P-value ^a^ |
| --- | --- | --- | --- |
| Number of cutaneous nf |  |  |  |
| 0 | 13 | 2 (2) | 0.012 (0.023) |
| ≥ 1 | 16 | 18 (16) |  |

a: two-tailed Fisher’s exact test. The P value in parenthesis was calculated for the comparison between the number of patients with cutaneous neurofibromas and type-1 *NF1* deletions of 1.4-Mb and the number of patients with cutaneous neurofibromas from the control cohort. Patients with atypical *NF1* deletions larger than 1.4-Mb were not included in this comparison.

# Supplementary Table S8:

Number of children with intragenic *NF1* mutations or *NF1* microdeletions in the age group of 1-3 years with or without plexiform neurofibromas (pnf) as analysed in this study.

|  | Number of children with intragenic *NF1* mutations | Number of children with *NF1* microdeletions | P-value ^a^ |
| --- | --- | --- | --- |
| with pnf | 1 (9%) | 8 (67%) | 0.0094 |
| without pnf | 10 (91%) | 4 (33%) |  |

a: two-tailed Fisher’s exact test

# Supplementary Table S9:

Total internal tumour volume in millilitres as determined by volumetric analysis in patients with and without *NF1* microdeletions. The total internal tumour volume in patients without *NF1* microdeletions has been determined in our previous study [39]. Patients with *NF1* microdeletions were analysed in the study presented here.

| NF1 patients without *NF1* microdeletions | | | Patients with *NF1* microdeletions | | |
| --- | --- | --- | --- | --- | --- |
| Patient ID | Age in years | Total internal tumour volume in ml | Patient ID | Age in years | Total internal tumour volume in ml |
| 860 | 3 | 0 | E-102 | 1 | 25 |
| 977 | 3 | 6 | 4904 | 2 | 90 |
| 953 | 4 | 116 | E-103 | 2 | 50 |
| 955 | 4 | 0 | E-101 | 3 | 801 |
| 997 | 5 | 335 | 4631 | 3 | 100 |
| 969 | 5 | 5 | 3415 | 4 | 100 |
| 610 | 10 | 0 | 2490 | 4 | 200 |
| 353 | 11 | 0 | 1261 | 7 | 57 |
| 688 | 11 | 496 | 4070 | 7 | 50 |
| 930 | 12 | 6 | 450 | 9 | 1850 |
| 405 | 12 | 0 | 867 | 9 | 90 |
| 918 | 12 | 0 | 4092 | 10 | 400 |
| 958 | 14 | 3 | 3751 | 10 | 400 |
| 967 | 14 | 41 | 3350 | 11 | 20 |
| 124 | 14 | 203 | 1333 | 12 | 2400 |
| 1284 | 15 | 18 | 1807 | 13 | 180 |
| 436 | 15 | 880 | E-105 | 14 | 800 |
| 158 | 16 | 31 | 1598 | 15 | 100 |
| 398 | 16 | 0 | 270 | 18 | 2500 |
| 837 | 16 | 168 | 3772 | 19 | 900 |
| 555 | 16 | 0 |  |  |  |
| 2003 | 16 | 0 |  |  |  |
| 896 | 16 | 14 |  |  |  |
| 404 | 17 | 688 |  |  |  |
| 485 | 17 | 141 |  |  |  |
| 797 | 18 | 0 |  |  |  |
| 890 | 18 | 0 |  |  |  |
| 600 | 18 | 2 |  |  |  |

# Supplementary Table S10:

Number of children with optic pathway glioma (OP glioma) in the group of children reported by Listernick et al. [74] and in the 30 children with *NF1* microdeletions investigated in this study. The children analysed by Listernick et al. [74] were not selected pertaining to *NF1* mutation type and hence represent the general population of children with NF1.

|  | Number of children studied by Listernick et al. [74] | Number of children with *NF1* microdeletions analysed here | P-value ^a^ |
| --- | --- | --- | --- |
| with OP glioma | 10 (15%) | 3 (10%) | 0.74 |
| without OP glioma | 55 | 26 |  |

a: two-tailed Fisher’s exact test

# Supplementary Table S11:

Skeletal anomalies, scoliosis, dysplasia or other tumours than brain tumours and neurofibromas as well as congenital heart defects in children with *NF1* microdeletions

| **ID** | **Age at investigation** | **Skeletal anomalies** | **Dysplasia or tumour** | **Congenital heart defect** | **Scoliosis^a^** |
| --- | --- | --- | --- | --- | --- |
| E-102 | 1 y | **−** | dysplasia of the vermis cerebelli | n.a. | **−** |
| 4904,0 | 2 y | **−** | **−** | **−** | + (10-15° Th) |
| 2483 | 2 y | pes cavus, both feet | **−** | **−** | + (20-30° Th) |
| 1333 | 2 y  9 y | bone cysts | **−** | **−** | + (15-20° Th/L, 2 y),  (>45° Th/L, surgery necessary at the age of 9 y) |
| 1598 | 2 y | pectus excavatum | dysplasia of the cerebellum | ventricular septal defect | **−** (<10° Th) |
| 4092 | 2 y | **−** | syringomyelia | atrial septal defect | **−** |
| 4070 | 2 y | dysplastic tibia and fibula of the left leg | **−** | n.a. | **−** (<10° Th) |
| 3415 | 2 y | **−** | microgyria | n.a. | + (10-15° Th) |
| E-103 | 2 y | **−** | **−** | n.a. | **−** |
| E-101 | 3 y | pectus excavatum | **−** | atrial septal defect with right bundle branch block, pulmonic stenosis | + (10-15° Th/L) |
| 4631 | 3 y | bone cysts | **−** | ventricular septal defect | **−** |
| 2490 | 3 y | pectus excavatum | **−** | hypertrophic cardiomyopathy | **−** |
| 3791 | 3 y | n.a. | n.a. | ventricular septal defect | **−** (< 10° Th) |
| 4061 | 4 y | pes cavus, one foot | **−** | n.a. | **−** |
| 442 | 4 y | pes cavus, one foot | **−** | **−** | + (>30° Th/L) |
| 4253 | 4 y | agenesis of the corpus callosum | **−** | pulmonic stenosis | + (>15° L) |
| 450 | 4 y | pectus excavatum | **−** | **−** | + (10° Th) |
| 1261 | 5 y | deformity of the intervertebral discs | **−** | **−** | + (>30° Th) |
| E-104 | 5 y | **−** | **−** | pulmonary valve stenosis | **−** |
| 284 | 6 y | pectus excavatum | **−** | n.a. | + (15° Th) |
| 1807 | 7 y | **−** | **−** | n.a. | **−** |
| DN56837 | 8 y | **−** | **−** | n.a. | **−** |
| 3350 | 8 y | pes cavus, one foot | mild dysplasia of the cerebellum | **−** | **−** (< 10° Th) |
| PFA | 9 y | pes cavus, both feet | **−** | n.a. | + (15° Th) |
| 3751 | 9 y | **−** | **−** | n.a. | **−** |
| 3772 | 11 y | pes cavus, one foot | **−** | n.a. | + (25-30° Th) |
| 867 | 12 y | pectus excavatum | **−** | **−** | + (10-15° Th/L) |
| E-105 | 14 y | **−** | aqueductal stenosis | aortic insufficiency | + (>15° Th) |
| 619/430 | 15 y | **−** | **−** | n.a. | + (20° Th/L) |
| 270 | 15 y | **−** | granular cell tumour of the lower jaw | **−** | + (10° L) |

a: the numbers in parentheses indicate the Cobb angle. A Cobb angle less than 10° was not considered as indicative of scoliosis. Th: thoracic; L: lumbar.

− : absent; + : present; n.a.: not analysed; y: year(s)

Supplementary Table S12: Education, developmental delay, cognitive abilities, autism and attention deficit hyperactivity disorder in the patients with *NF1* microdeletions investigated in the present study. n.a.: not analysed.

| **ID** | **Sex** | **Deletion** | **Education** | **Developmental delay in motor skills and speech^a^** | **FSIQ^b^** | **T-score^c^** | **Occupation** | **ADHD^d^** |
| --- | --- | --- | --- | --- | --- | --- | --- | --- |
| E-102 | m | 1.4-Mb | preschool [1 y] | + | n.a. | n.a. | n.a. | n.a. |
| 4904,0 | m | 1.4-Mb | preschool [2 y] | + | n.a. | n.a. | n.a. | n.a. |
| 2483 | m | 4.7-Mb | school for children with physical and cognitive disabilities | + | 69 | 78 | workshop for the disabled,  died at the age of 26 y | n.a. |
| 1333 | f | 1.4-Mb | school for children with learning disabilities | + | 70 | 73 | died at the age of 13 y | + |
| 1598 | m | 1.4-Mb | general certificate of secondary education (Realschule) | + | 90 | 63 | apprenticeship as assistant for social education workers | + |
| 4092 | f | 1.4-Mb | student with special needs in inclusion education | + | 73 | 68 | n.a. | + |
| 3415 | m | 1.4-Mb | school for students with cognitive disabilities | + | 51 | 61 | not occupied | + |
| E-103 | m | 1.4-Mb | preschool [2 y] | + | n.a. | n.a. | n.a. | n.a. |
| E-101 | m | 1.4-Mb | preschool [3 y] | + | n.a. | 66 |  | n.a. |
| 4631 | f | 1.4-Mb | general certificate of secondary education (Realschule) | + | 85 | 56 | nurse for the elderly | + |
| 2490 | f | 1.4-Mb | student with special needs in inclusion education; special school for language therapy | + | 88 | 51 | n.a. | n.a. |
| 3791 | m | 1.4-Mb | student with special needs in inclusion education | + | n.a. | 68 | not occupied | n.a. |
| 4061 | m | 1.4-Mb | student with special needs in inclusion education | + | 80 | 55 | n.a. | + |
| 442 | m | 2-Mb | student with special needs in inclusion secondary education (Hauptschule and Realschule) | + | 76 | 70 | not occupied | + |
| 4253 | f | 1.4-Mb | student with special needs in inclusion education | + | 80 | 66 | n.a. | + |
| 450 | f | 1.4-Mb | student with special needs in inclusion secondary education (Hauptschule), special school for children with learning disabilities | + | 65 | 64 | regularly employed as kitchen aid in a coffee bar | + |
| 1261 | m | 1.4-Mb | secondary school | − | 110 | 63 | college student | n.a. |
| E-104 | m | 1.4-Mb | preschool [3 y] | + | n.a. | 67 | n.a. | n.a. |
| 284 | f | 1.4-Mb | school for children with learning disabilities | + | 91 | n.a. | n.a. | ̶ |
| 1807 | m | 1.4-Mb | secondary school leaving certificate acquired in a school for persons with learning disabilities | + | 75 | 51 | apprentice in professional training | n.a. |
| DN56837 | f | 1.4-Mb | special school for children with learning disabilities | + | 69 | n.a. | n.a. | n.a. |
| 3350 | m | 1.4-Mb | secondary modern school | + | 84 | 63 | n.a. | + |
| 4070 | f | 1.4-Mb | student with special needs in inclusion education in a regular school | + | 76 | 64 | n.a. | + |
| PFA | m | 1.4-Mb | school for children with learning disabilities | + | 70 | 62 | workshop for the disabled | n.a. |
| 3751 | f | 1.4-Mb | general certificate of secondary education (Realschule) | − | 97 | 56 | economy school (Wirtschaftsschule) [13 y] | + |
| 3772 | m | 1.4-Mb | general certificate of secondary education (Realschule) | + | 92 | 79 | specialist for warehouse logistics | + |
| 867 | f | 1.4-Mb | secondary education (Realschule) | + | 78 | n.a. | office clerk | + |
| E-105 | m | 1.4-Mb | school for children with learning disabilities | + | 70 | 69 | workshop for the disabled | + |
| 619/430 | f | 3-Mb | school for children with physical and cognitive disabilities | + | 70 | 75 | workshop for the disabled | n.a. |
| 270 | m | 1.4-Mb | student with special needs in inclusion education in a regular school; certificate of secondary modern school education (Hauptschule) | + | 56 | 63 | not occupied, died at the age of 27 y | ̶ |

a: Global developmental delay in motor and language skills was assessed by means of the modified Munich Functional Developmental Diagnostics (Münchener Funktionelle Entwicklungsdiagnostik, MFED) for children at the age of 1-3 years [40, 41].

b: Full-scale intelligence quotient (FSIQ). Cognitive ability in the patients with *NF1* microdeletions was assessed at the age of 6-18 years by means of the German language versions of Wechsler Intelligence Scales (Hamburg-Wechsler Intelligence Scales HAWIK-R, HAWIK-III and HAWIK-IV) [42, 43]. Based on these tests, mean full-scale IQ (FSIQ) values were determined.

c: Autism spectrum disorder (ASD) assessed by the T-scores according to the Social Responsiveness Scale questionnaire (SRS).

d: Attention deficit hyperactivity disorder (ADHD) was assessed as described in our previous study [18].
+: present; ̶: absent

**Supplementary Table S13:**

Comparison of the frequency of autistic symptoms in patients with type-1 *NF1* microdeletions analysed here and NF1 patients reported previously [93, 105]. The patients investigated by Eijk et al. [93] and Morris et al. [105] were not selected pertaining to *NF1* mutation type and thus represent the general NF1 population. Autistic symptoms were assessed by means of T-scores obtained by SRS questionnaires. The three patients with atypical *NF1* deletions were not included.

|  | **Morris et al. [105]** | **Eijk et al. [93]** | **This study** | **P-value^a^** | **P-value^b^** |
| --- | --- | --- | --- | --- | --- |
| Total number of patients analysed | 531 | 103 | 21 |  |  |
| T-score ≥ 76 | 70 (13.2%) | 9 (8.7%) | 1 (4.8%) |  |  |
| T-score ≥ 60 − 75 | 138 (26.0%) | 17 (16.5%) | 15 (71.4%) | 1.7 x 10^-6^ **^c^** | 6.4 x 10^-5^ **^c^** |
| T-score < 60 | 323 (60.8%) | 77 (74.8%) | 5 (23.8%) |  |  |
| Mean total T-score [SD] | 58.2 [13.4] | 54.7 [12.6] | 63.2 [6.6] | 0.0033**^d^** | 0.0896**^d^** |

a: Eijk et al. [93] vs. this study

b: Morris et al. [105] vs. this study

c: two-sided Fisher’s exact test

d: two-sided unpaired *t*-test

SD: standard deviation

# **Supplementary Table S14**:

Comparison of the T-scores in female versus male patients with *NF1* microdeletions analysed in this study.

|  | **Female patients with *NF1* microdeletions** | **Male patients with *NF1* microdeletions** | **P-value^a^** |
| --- | --- | --- | --- |
| Number of patients analysed | 9 | 15 |  |
| T-score ≥ 76 | 0 | 2 |  |
| T-score ≥ 60-75 | 6 | 11 | 0.6**^a^** |
| T-score < 60 | 3 | 2 |  |
| Mean total T-score [SD] | 63.66 [6.22] | 65.2 [5.41] | 0.5**^b^** |

a: two-tailed Fisher’s exact test

b: two-sided unpaired *t*-test

SD: standard deviation
